# Supplementary material for: Identification of photoperiod-induced specific miRNAs in the adrenal glands of Sunite sheep (Ovis aries)
Source: Front Vet Sci. 2022 Jul 22;9:888207. doi: 10.3389/fvets.2022.888207 (PMC9354845; doi:10.3389/fvets.2022.888207)
Supplement: Supplementary file 1 [file Data_Sheet_1.ZIP › Supplementary file/miRDeep2 file.zip!/miRDeep2 file/Know_Structure/SP42A2/oar-mir-136.pdf]

miRBase precursor	: oar-mir-136
Total read count	: 9993
oar-miR-136 read count	: 2528
remaining reads	: 7465

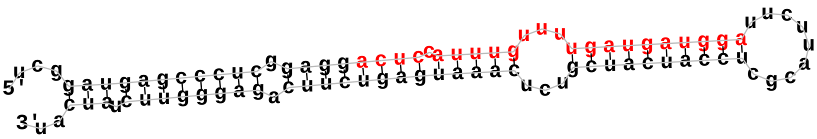

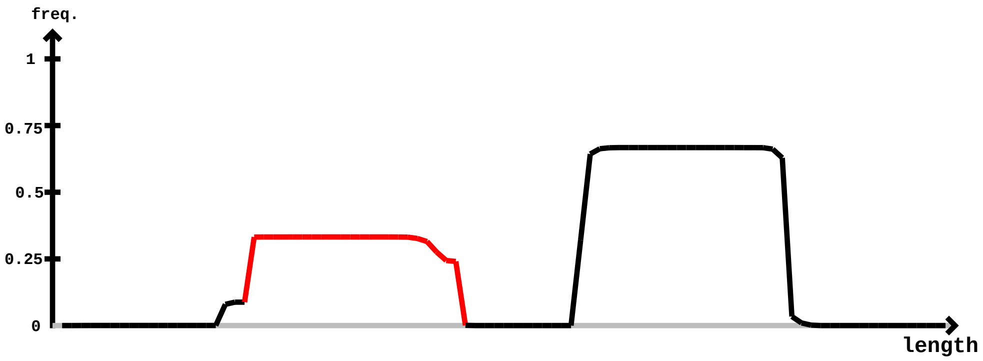

oar-miR-136

```
5'- ucggaugagcccucggaggacuccauuuguuuugaugauggauucuuacgcuccaucaucgucucaaaugagucuucagaggguucuaucau   -3'    exp
    ...(((((((((((.((((((((.(((((...((((((((((.........))))))))))...))))))))))))).)))))))).)))..    reads   mm   sample
    .cggaugagcccucggagg.........................................................................    2       0    seq
    ..ggaugagcccucggagg.........................................................................    1       0    seq
    ................aggacuccauuuguuuug..........................................................    2       0    seq
    ................aggacuccauuuguuuuga.........................................................    5       0    seq
    ................aggacuccauuuguuuugau........................................................    49      0    seq
    ................aggacuccauuuguuuugaug.......................................................    114     0    seq
    ................aggacuccauuuguuuugauga......................................................    376     0    seq
    ................aggacuccauuuguuuugaugau.....................................................    247     0    seq
    .................ggacuccauuuguuuugau........................................................    1       0    seq
    .................ggacuccauuuguuuugaug.......................................................    1       0    seq
    .................ggacuccauuuguuuugauga......................................................    17      0    seq
    .................ggacuccauuuguuuugaugau.....................................................    62      0    seq
    ...................acuccauuuguuuugaugau.....................................................    8       0    seq
    ...................Ccuccauuuguuuugaugaug....................................................    3       1    seq
    ...................acuccauuuguuuugaugaug....................................................    32      0    seq
    ...................acuccauuuguuuugaugaugg...................................................    2392    0    seq
    ...................acuccauuuguuuugaugaugga..................................................    8       0    seq
    ....................cuccauuuguuuugaugau.....................................................    1       0    seq
    ....................cuccauuuguuuugaugaugg...................................................    1       0    seq
    ....................cuccauuuguuuugaugaugA...................................................    1       1    seq
    .......................cauuuguuuugaugaugga..................................................    1       0    seq
    .....................................................caucaucgucucaaaug......................    2       0    seq
    .....................................................caucaucgucucaaaugag....................    2       0    seq
    .....................................................caucaucgucucaaaugagu...................    40      0    seq
    .....................................................caucaucgucucaaaugaguc..................    236     0    seq
    .....................................................caucaucgucucaaaugagucu.................    2904    0    seq
    .....................................................caucaucgucucaaaugagucuu................    20      0    seq
    ......................................................aucaucgucucaaaugagC....................    5       1    seq
    ......................................................aucaucgucucaaaugagu...................    8       0    seq
    ......................................................aucaucgucucaaaugaguc..................    89      0    seq
    ......................................................aucaucgucucaaaugagucu.................    2972    0    seq
    ......................................................aucaucgucucaaaugagucuu................    145     0    seq
    ......................................................aucaucgucucaaaugagucuuc...............    6       0    seq
    .......................................................ucaucgucucaaaugagu...................    1       0    seq
    .......................................................ucaucgucucaaaugaguc..................    1       0    seq
```

oar-miR-136

```
ucggaugagcccucggaggacuccauuuguuuugaugauggauucuuacgcuccaucaucgucucaaaugagucuucagaggguucuaucau
.......................................................ucaucgucucaaaugagucu.................	63	0	seq
.......................................................ucaucgucucaaaugagucuu................	72	0	seq
.......................................................ucaucgucucaaaugagucuuc...............	52	0	seq
.......................................................ucaucgucucaaaugagucuuca..............	8	0	seq
........................................................caucgucucaaaugagucuu................	4	0	seq
........................................................caucgucucaaaugagucuC................	1	1	seq
........................................................caucgucucaaaugagucuuc...............	22	0	seq
........................................................caucgucucaaaugagucuuca..............	9	0	seq
.........................................................aucgucucaaaugagucu.................	6	0	seq
.........................................................aucgucucaaaugagucuuc...............	1	0	seq
```
